# Adverse pregnancy outcomes associated with advanced maternal age among Libyan women: a cross-sectional study at Benghazi medical centre

**DOI:** 10.3389/frph.2026.1965640

**Published:** 2026-10-12

**Authors:** Milad Gahwagi, Wud Abdulali, Entesar Elmajrysy

**Affiliations:** 1 Faculty of Medicine, Benghazi Medical University, Benghazi, Libya; 2 Benghazi Medical Centre, Benghazi, Libya; 3 Faculty of Medicine, Libyan International University, Benghazi, Libya

**Keywords:** advanced maternal age, caesarean section, Libya, neonatal outcomes, pregnancy outcomes, preterm birth

## Abstract

**Introduction:**

Advanced maternal age (AMA), commonly defined as maternal age ≥35 years, has been associated with adverse pregnancy outcomes. However, evidence from Libya and the wider North African region remains limited. This study examined maternal and neonatal outcomes associated with AMA in a Libyan cohort.

**Methods:**

This retrospective, record-based cross-sectional study included deliveries at Benghazi Medical Centre between 1 April 2017 and 25 December 2019. A systematic random sample of 450 singleton delivery records was selected from 9,450 eligible deliveries. After excluding six women aged 17–19 years, the final analysis included 444 women aged 20–45 years. Firth penalized-likelihood logistic regression was used to adjust for parity, body mass index, and pre-existing diabetes mellitus.

**Results:**

Of the 444 women included, 243 (54.7%) were aged ≥35 years. AMA was associated with higher crude odds of preterm labour (OR 2.01, 95% CI 1.09–3.68) and preterm birth (OR 2.08, 95% CI 1.12–3.86). After adjustment, these associations were attenuated, and the confidence intervals included the null (aOR 1.76, 95% CI 0.93–3.31 and aOR 1.87, 95% CI 0.98–3.56, respectively). Pre-existing diabetes was observed only among women aged ≥35 years. Meconium aspiration was less frequent in the AMA group, although this finding should be interpreted cautiously because of the small number of events.

**Discussion:**

In this predominantly multiparous Libyan cohort from a tertiary referral centre, adjustment for baseline maternal characteristics reduced the strength of the main crude associations with maternal age. These findings suggest that maternal age should be considered alongside parity and maternal medical history when assessing antenatal risk.

## 1 Introduction

Pregnancy at age 35 years or older, commonly referred to as advanced maternal age (AMA), has become increasingly prevalent worldwide (1). Delayed childbearing reflects demographic and social changes including prolonged education, career development, changing reproductive preferences, and greater availability of assisted reproductive technologies (2).

Advancing maternal age is accompanied by reproductive, vascular, metabolic, and placental changes that may increase obstetric risk. Older women also have a higher prevalence of chronic conditions such as hypertension, type II diabetes mellitus, and obesity. Previous studies have associated AMA with preterm birth, pregnancy-associated hypertension (3), placental complications, caesarean section, fetal loss, low birth weight, and neonatal morbidity, although the magnitude of these associations varies according to parity, comorbidities, healthcare access, and obstetric practice (4).

Systematic reviews, meta-analyses, and large observational studies support increased risks of selected maternal and neonatal complications with advancing maternal age (5–7). However, risks are heterogeneous across populations. Large multicountry studies emphasize the importance of controlling for maternal and facility-level characteristics (4), while Middle Eastern studies have reported associations with preterm delivery, gestational diabetes, and caesarean delivery after adjustment for relevant covariates (8–10). These regional findings are particularly relevant because older mothers in Middle Eastern populations may frequently be multiparous rather than predominantly nulliparous (8, 10).

Maternal age is correlated with parity, previous obstetric exposure, metabolic disease, and previous caesarean delivery. Consequently, crude comparisons may overestimate or obscure age-related associations, making adjustment for clinically important maternal characteristics necessary.

Few studies in the published Libyan literature have investigated AMA or maternal and neonatal outcomes. It is possible that results from studies conducted in other countries may be less applicable to Libya because of differences in antenatal care utilization, referral systems, parity, background morbidity, and access to specialist care.

Benghazi Medical Centre is a public tertiary referral institution serving Benghazi and eastern Libya. The period from April 1, 2017, through December 25, 2019, was selected because consistently structured delivery records were available throughout this period. While historical hospital data cannot provide estimates of the current national prevalence of AMA, they allow age-related trends to be examined in a previously understudied Libyan context.

Maternal, obstetric, fetal, and neonatal outcomes were examined across age groups (20–29, 30–34, 35–39, and 40–45 years). The four age categories allow differentiation among younger reproductive ages, the period immediately before the traditional AMA cutoff point, traditional AMA, and very advanced reproductive age while maintaining a sufficient number of cases within each category. Additionally, maternal age was examined continuously in the sensitivity analysis. Based on prior research, it was expected that certain adverse outcomes would occur more frequently among those aged ≥35 years than among those aged <35 years; however, it was anticipated that some of these crude associations might diminish or disappear after adjustment for parity and other clinically relevant maternal factors.

## 2 Methods

### 2.1 Study design and setting

This retrospective, record-based cross-sectional study was conducted in the Department of Obstetrics and Gynecology at Benghazi Medical Centre, Benghazi, Libya. Benghazi Medical Centre is a public, university-affiliated tertiary referral center that provides both low- and high-risk obstetric services and receives referrals for obstetric care from Benghazi and eastern Libya. Therefore, the study population is better described as a sample drawn from a tertiary hospital population rather than an epidemiologically representative sample of all pregnancies in Libya.

Delivery records from 1 April 2017 to 25 December 2019 were examined. This period was selected because complete and consistently structured maternal and neonatal delivery records were available throughout the interval.

### 2.2 Participants, sampling, and data cleaning

A total of 9,612 deliveries to Libyan women were recorded during the study period. Of these, 162 multiple-gestation deliveries were excluded from the sampling frame because multiple pregnancy independently affects gestational age, fetal growth, delivery mode, and neonatal morbidity. The remaining 9,450 singleton deliveries constituted the sampling frame.

Systematic random sampling was used to select 450 records. The sampling interval was 9,450/450 = 21. After chronological ordering of the singleton deliveries and selection of a random starting position between 1 and 21, every 21st record was selected until 450 records were obtained.

Eligible records were deliveries to Libyan women at Benghazi Medical Centre between 1 April 2017 and 25 December 2019 that involved a singleton pregnancy and had retrievable maternal and delivery records. Multiple gestations were excluded before construction of the sampling frame. The prespecified primary analysis was restricted to maternal age 20–45 years; therefore, six sampled women aged 17–19 years were excluded after sampling. No sampled women were older than 45 years, and no further records were excluded after data cleaning.

The final analytic sample therefore comprised 444 women. Participant flow was 9,612 recorded deliveries, of which 9,450 were singleton deliveries; 450 records were systematically sampled, resulting in 444 women aged 20–45 years.

Detailed socioeconomic indicators beyond employment status, including education, household income, and insurance status, were not systematically available. Therefore, the socioeconomic representativeness of the cohort could not be established.

Data quality was assessed before analysis. Variables were checked for completeness, biological plausibility, internal consistency, and agreement with source clinical records when discrepancies were identified. A 10% random subsample was independently rechecked against the original records. Logical consistency checks were applied to maternal age, parity, gestational age at labour and delivery, birth weight, mode of delivery, and fetal/neonatal mortality classifications. No outcome data were imputed.

### 2.3 Variables and definitions

Data were abstracted from the contemporaneous maternal and neonatal clinical records. Maternal age, employment status, BMI, parity, presenting symptoms, gestational age, haemoglobin, birth weight, Apgar scores, and mode of delivery were transcribed from the recorded clinical fields. Preeclampsia, antepartum haemorrhage, postpartum haemorrhage, meconium aspiration, shoulder dystocia, NICU admission, and other clinical outcomes were classified according to the diagnosis or event documented in the source record. The component measurements required to retrospectively re-adjudicate each clinical diagnosis were not consistently available; therefore, these diagnoses were not independently reclassified.

Maternal age was analysed using the four age categories and a secondary binary classification of <35 vs. ≥35 years. Employment status was classified as employed or unemployed. BMI categories were retained as recorded in the source dataset.

Parity was categorized as primiparity vs. multiparity. Parity was considered an important potential confounding factor because its prevalence differed substantially across age groups. In addition, parity has been shown to be independently associated with obstetric outcomes. Because the exact number of previous deliveries was not consistently recorded, grand multiparity could not be assessed.

Delivery mode was categorized into one of four categories: spontaneous vaginal delivery (SVD), induced vaginal delivery (IVD), elective cesarean section (ECS), and emergency cesarean section (EmCS). Regardless of whether delivery was by ECS or EmCS, all patients who delivered by cesarean section were included in the overall cesarean delivery category. Prior cesarean delivery status was not uniformly recorded and therefore could not be included as a covariate.

Preterm labour was defined as onset of labour before 37 completed weeks, whereas preterm birth referred to delivery before 37 completed weeks. The outcomes were analysed separately because they are clinically related but not interchangeable. Preterm labour could be followed by either preterm or term delivery, whereas preterm birth could also occur without preceding recorded preterm labour, including medically indicated delivery. Their patient-level overlap was therefore examined to clarify the relationship between the two outcomes.

Low birth weight was defined as <2,500 g. Birth weight was also analysed continuously and fetal overgrowth was assessed using both >4,000 g and >4,500 g. The >4,500-g threshold reflected the definition of marked macrosomia in the original clinical dataset, whereas >4,000 g was **used** for comparability with international obstetric literature.

An Apgar score <7 was classified as low. Pre-existing diabetes was treated as a baseline maternal medical condition. Gestational diabetes mellitus was not reliably recorded as a distinct variable and could not be analysed separately.

Mortality variables were reconciled using standardized temporal definitions. Antepartum intrauterine fetal death (IUFD) referred to fetal death before labour at ≥28 completed weeks. Stillbirth referred to birth without signs of life at ≥28 completed weeks and included antepartum IUFD and intrapartum fetal death. Early neonatal death referred to death of a liveborn infant within the first seven completed days. Perinatal death comprised stillbirth plus early neonatal death, while neonatal death referred to death during the first 28 completed days. IUFD was therefore treated as a subset of stillbirth rather than an additional independent event. Congenital structural abnormalities and chromosomal anomalies were not systematically available and were not analysed.

### 2.4 Statistical analysis

Categorical variables are presented as counts and percentages. Continuous birth weight is presented as mean ± standard deviation. Four-group categorical comparisons were assessed using Pearson's chi-square omnibus tests; because some tables contained sparse cells, these omnibus comparisons were interpreted as exploratory. Significant omnibus tests were followed by pairwise comparisons using Pearson's chi-square test, Fisher's exact test, or the Fisher–Freeman–Halton exact test as appropriate, with Bonferroni correction for the six possible pairwise comparisons. In the secondary comparison of women aged ≥35 vs. <35 years, crude two-group *p*-values were obtained using Fisher's exact test. One-way analysis of variance compared mean birth weight across age groups.

A secondary binary analysis compared women aged ≥35 years with those aged <35 years. Crude odds ratios (ORs) and 95% confidence intervals (CIs) were calculated. A directed acyclic graph (DAG) was specified to organize hypothesized relationships among maternal age, measured maternal characteristics, incompletely measured reproductive and socioeconomic factors, and maternal/neonatal outcomes (Figure 1) (11). Maternal age was the exposure; parity, BMI category, and pre-existing diabetes were the measured covariates selected *a priori* for principal adjusted models. Detailed socioeconomic characteristics, antenatal-care exposure, previous preterm birth, and previous caesarean delivery were incompletely measured or unavailable.

**Figure 1:**
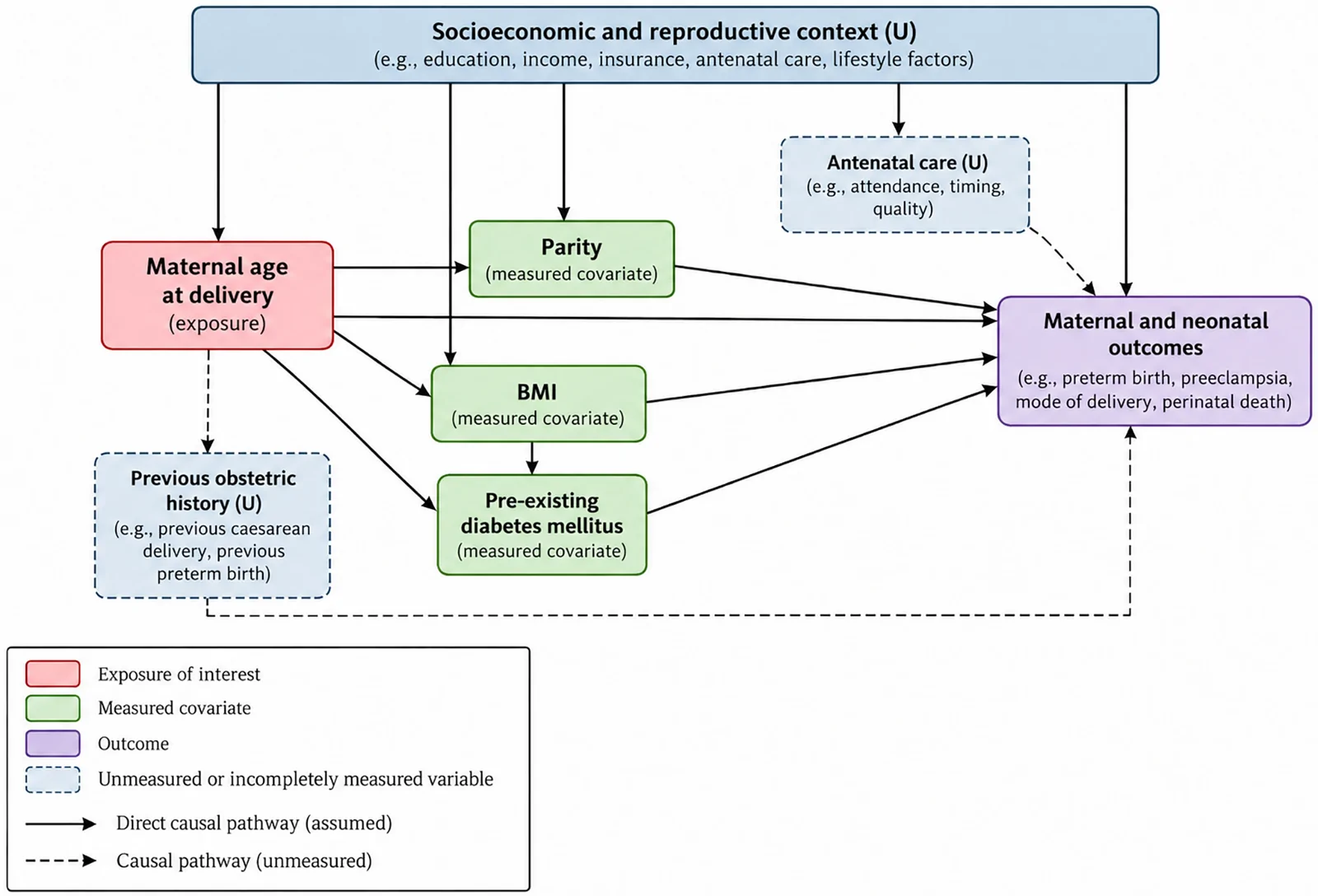
Directed acyclic graph (DAG) describing the hypothesized relationships between maternal age, measured covariates, incompletely measured characteristics, and maternal and neonatal outcomes. Maternal age at delivery was the exposure of interest. Parity, BMI, and pre-existing diabetes mellitus were measured covariates. Socioeconomic and reproductive context, antenatal-care characteristics, previous caesarean delivery, and previous preterm birth were incompletely measured or unavailable. Solid arrows indicate assumed direct pathways, and dashed arrows indicate pathways involving incompletely measured variables.

Because maternal age was strongly associated with parity and several outcomes were uncommon, adjusted associations were estimated using Firth penalized-likelihood logistic regression, which reduces small-sample bias and problems related to separation but does not eliminate imprecision caused by sparse data. To avoid reporting unstable multivariable estimates, outcomes with any exposure-by-outcome cell count <5 were not subjected to adjusted regression and were reported using raw cell counts and crude associations only. Firth models were retained for the principal outcomes and other outcomes without cells <5 and were adjusted *a priori* for parity, BMI category, and pre-existing diabetes mellitus based on the DAG. Previous caesarean delivery, previous preterm birth, gestational diabetes, and detailed socioeconomic characteristics could not be included because they were not consistently available.

Multicollinearity was assessed using variance inflation factors (VIFs). All VIFs were <2.00, indicating no problematic multicollinearity. Results are presented as adjusted odds ratios (aORs), 95% confidence intervals (CIs), and two-tailed *p*-values. Sensitivity analyses included modeling maternal age as a continuous variable. Interpretation emphasized the effect estimates, CIs, event frequencies, and clinical relevance, in addition to *p*-values. A result was considered statistically significant when the two-tailed *p*-value was <.05 in the primary analyses. Microsoft Excel was used for data cleaning, and R statistical software was used for statistical analysis**.**

### 2.5 Ethical considerations

Ethical approval was obtained from the Institutional Research Ethics Committee/Institutional Review Board of Benghazi Medical Centre on 3 January 2017 (IRB approval No. Jan/3). The study was conducted in accordance with the Declaration of Helsinki and applicable Libyan regulations. Because the study involved retrospective analysis of anonymized routinely collected clinical records, the requirement for individual informed consent was waived by the Institutional Research Ethics Committee/Institutional Review Board. Data were anonymized before analysis.

## 3 Results

### 3.1 Study population

Of 9,612 deliveries to Libyan women, 9,450 singleton deliveries constituted the sampling frame. Systematic random sampling yielded 450 records, and exclusion of six women aged 17–19 years resulted in 444 women in the primary analysis.

The age distribution was 137 (30.9%) aged 20–29 years, 64 (14.4%) aged 30–34 years, 174 (39.2%) aged 35–39 years, and 69 (15.5%) aged 40–45 years. Overall, 243 women (54.7%) were aged ≥35 years. The high proportion of AMA reflects this tertiary-referral cohort and should not be interpreted as the prevalence of AMA in the general Libyan obstetric population.

### 3.2 Baseline and presentation characteristics

Parity differed across age groups (overall *p* < 0.001), with multiparity increasing from 72.3% among women aged 20–29 years to 89.1% among women aged 35–39 years and 88.4% among those aged 40–45 years (Table 1). After Bonferroni correction, the difference between women aged 20–29 and 35–39 years remained significant (adjusted *p* = 0.0008).

**Table 1:** Baseline maternal, medical, and presentation characteristics by maternal age group.

Characteristic	20–29 years (*n* = 137)	30–34 years (*n* = 64)	35–39 years (*n* = 174)	40–45 years (*n* = 69)	*p* value
Employed	13 (9.5%)	3 (4.7%)	15 (8.6%)	12 (17.4%)	0.081
Unemployed	124 (90.5%)	61 (95.3%)	159 (91.4%)	57 (82.6%)	0.081
Primiparous	38 (27.7%)	13 (20.3%)	19 (10.9%)	8 (11.6%)	<0.001
Multiparous	99 (72.3%)	51 (79.7%)	155 (89.1%)	61 (88.4%)	<0.001
BMI: normal weight	55 (40.1%)	29 (45.3%)	71 (40.8%)	23 (33.3%)	0.800
BMI: overweight	76 (55.5%)	33 (51.6%)	98 (56.3%)	42 (60.9%)	0.800
BMI: obesity	6 (4.4%)	2 (3.1%)	5 (2.9%)	4 (5.8%)	0.800
Pre-existing diabetes mellitus	0 (0.0%)	0 (0.0%)	3 (1.7%)	4 (5.8%)	0.011
Abnormal respiratory findings	0 (0.0%)	0 (0.0%)	0 (0.0%)	2 (2.9%)	0.012
Abnormal cardiovascular findings	0 (0.0%)	1 (1.6%)	0 (0.0%)	0 (0.0%)	0.114
Haemoglobin at admission <8 g/dL	2 (1.5%)	2 (3.1%)	1 (0.6%)	1 (1.4%)	0.718
Haemoglobin at admission 8–10.5 g/dL	46 (33.6%)	22 (34.4%)	52 (29.9%)	19 (27.5%)	0.718
Haemoglobin at admission >10.5 g/dL	88 (64.2%)	40 (62.5%)	121 (69.5%)	48 (69.6%)	0.718
No presenting complaint	103 (75.2%)	51 (79.7%)	149 (85.6%)	62 (89.9%)	0.026
Leaking liquor/fluid	33 (24.1%)	13 (20.3%)	22 (12.6%)	6 (8.7%)	0.026
Per-vaginal bleeding	0 (0.0%)	0 (0.0%)	3 (1.7%)	1 (1.4%)	0.026
Pain at presentation	57 (41.6%)	31 (48.4%)	72 (41.4%)	34 (49.3%)	0.556

Values are *n* (% within age group). Baseline maternal medical characteristics are presented separately from pregnancy outcomes. For multicategory variables, the *p* value represents the overall comparison across maternal-age groups. *Post-hoc* comparisons following significant omnibus tests were interpreted using Bonferroni correction. Admission haemoglobin was unavailable for two women (one aged 20–29 years and one aged 40–45 years), and the primary presenting symptom was undocumented for one woman aged 20–29 years. Percentages are based on the full age-group denominators; statistical comparisons used available observations. Pain at presentation was recorded separately and could coexist with another presenting symptom.

Employment status and BMI did not differ significantly. Presenting symptoms differed overall (*p* = 0.026); *post-hoc* differences remained significant for 20–29 vs. 35–39 years (adjusted *p* = 0.0419) and 20–29 vs. 40–45 years (adjusted *p* = 0.0252). Pre-existing diabetes occurred in seven women, all aged ≥35 years. Abnormal respiratory findings occurred in only two women. Because these outcomes were sparse, their distributions were interpreted cautiously rather than from *p*-values alone.

### 3.3 Maternal complications and delivery outcomes

Preeclampsia, antepartum haemorrhage, perineal tear, postpartum haemorrhage, and postpartum haemoglobin did not differ significantly across age groups (Table 2).

**Table 2:** Obstetric and maternal delivery outcomes by maternal age group.

Characteristic	20–29 years (*n* = 137)	30–34 years (*n* = 64)	35–39 years (*n* = 174)	40–45 years (*n* = 69)	*p* value
Gestational age at onset of labour <32 weeks	3 (2.2%)	1 (1.6%)	3 (1.7%)	1 (1.4%)	0.337
Gestational age at onset of labour 32–36 weeks	9 (6.6%)	4 (6.2%)	25 (14.4%)	9 (13.0%)	0.337
Gestational age at onset of labour ≥37 weeks	125 (91.2%)	59 (92.2%)	146 (83.9%)	59 (85.5%)	0.337
Preeclampsia	2 (1.5%)	1 (1.6%)	5 (2.9%)	3 (4.3%)	0.589
Antepartum haemorrhage	4 (2.9%)	4 (6.2%)	6 (3.4%)	2 (2.9%)	0.663
Perineal tear	18 (13.1%)	9 (14.1%)	33 (19.0%)	12 (17.4%)	0.530
Postpartum haemorrhage	3 (2.2%)	4 (6.2%)	2 (1.1%)	1 (1.4%)	0.121
Postpartum haemoglobin <8 g/dL	2 (1.5%)	1 (1.6%)	2 (1.1%)	0 (0.0%)	0.772
Postpartum haemoglobin 8–10.5 g/dL	84 (61.3%)	39 (60.9%)	96 (55.2%)	37 (53.6%)	0.772
Postpartum haemoglobin >10.5 g/dL	51 (37.2%)	24 (37.5%)	76 (43.7%)	32 (46.4%)	0.772

Values are *n* (% within age group). Gestational age at onset of labour and gestational age at delivery were treated as distinct variables because preterm labour does not invariably result in immediate preterm birth.

Preterm labour before 37 weeks occurred in 17 of 201 women aged <35 years (8.5%) and 38 of 243 women aged ≥35 years (15.6%). The crude OR was 2.01 (95% CI 1.09–3.68); after adjustment for parity, BMI, and pre-existing diabetes, the estimate attenuated to aOR 1.76 (95% CI 0.93–3.31; *p* = 0.081).

Mode of delivery differed in the four-group omnibus analysis (*p* = 0.015; Table 3), but no pairwise comparison for the complete four-category delivery distribution remained significant after Bonferroni adjustment. When any caesarean delivery was analysed separately, the 30–34 vs. 35–39-year comparison remained significant (adjusted *p* = 0.0284).

**Table 3:** Mode of delivery by maternal age group.

Characteristic	20–29 years (*n* = 137)	30–34 years (*n* = 64)	35–39 years (*n* = 174)	40–45 years (*n* = 69)	*p* value
Mode of delivery, overall					0.015
Spontaneous vaginal delivery	114 (83.2%)	43 (67.2%)	144 (82.8%)	49 (71.0%)	**—**
Induced vaginal delivery	0 (0.0%)	1 (1.6%)	4 (2.3%)	1 (1.4%)	**—**
Elective caesarean section	5 (3.6%)	4 (6.2%)	7 (4.0%)	9 (13.0%)	**—**
Emergency caesarean section	18 (13.1%)	16 (25.0%)	19 (10.9%)	10 (14.5%)	**—**
Any caesarean section	23 (16.8%)	20 (31.2%)	26 (14.9%)	19 (27.5%)	0.010

Values are *n* (% within age group). The *p* = 0.015 value represents a single omnibus comparison of the complete four-category mode-of-d*e*livery distribution and should not be interpreted as a separate test for each delivery mode. None of the six pairwise age-group comparisons remained statistically significant after Bonferroni correction (pairwise *α* = 0.0083).

Any caesarean delivery occurred in 21.4% of women aged <35 years and 18.5% aged ≥35 years and was not associated with AMA after adjustment (aOR 0.91, 95% CI 0.55–1.50; *p* = 0.710). Emergency caesarean delivery was similarly not independently associated with AMA (aOR 0.70, 95% CI 0.40–1.22; *p* = 0.208). Interpretation remains limited by unavailable previous-caesarean history.

### 3.4 Fetal and neonatal outcomes

Mean birth weight and the proportion of neonates weighing >4,000 g did not differ significantly among age groups (*p* = 0.283 and *p* = 0.857, respectively; Table 4). Only three neonates weighed >4,500 g, limiting inference regarding marked macrosomia.

**Table 4:** Fetal and neonatal outcomes by maternal age group.

Characteristic	20–29 years (*n* = 137)	30–34 years (*n* = 64)	35–39 years (*n* = 174)	40–45 years (*n* = 69)	*p* value
Gestational age at birth <32 weeks	3 (2.2%)	1 (1.6%)	3 (1.7%)	1 (1.4%)	0.257
Gestational age at birth 32–36 weeks	9 (6.6%)	3 (4.7%)	25 (14.4%)	8 (11.6%)	0.257
Term birth ≥37 weeks	125 (91.2%)	60 (93.8%)	146 (83.9%)	60 (87.0%)	0.257
Birth weight, mean ± SD, g	3,218 ± 518	3,295 ± 522	3,186 ± 546	3,142 ± 571	0.283
Low birth weight <2,500 g	12 (8.8%)	3 (4.7%)	24 (13.8%)	5 (7.2%)	0.130
Birth weight >4,000 g	10 (7.3%)	6 (9.4%)	11 (6.3%)	5 (7.2%)	0.884
Marked macrosomia >4,500 g	1 (0.7%)	2 (3.1%)	0 (0.0%)	0 (0.0%)	0.061
Meconium aspiration	9 (6.6%)	5 (7.8%)	3 (1.7%)	1 (1.4%)	0.042
Shoulder dystocia	0 (0.0%)	1 (1.6%)	1 (0.6%)	0 (0.0%)	0.431
NICU admission	2 (1.5%)	3 (4.7%)	10 (5.7%)	4 (5.8%)	0.263
Antepartum IUFD ≥28 weeks	2 (1.5%)	2 (3.1%)	2 (1.1%)	2 (2.9%)	0.657
Stillbirth ≥28 weeks	2 (1.5%)	3 (4.7%)	2 (1.1%)	2 (2.9%)	0.328
Early neonatal death, 0–6 days	2 (1.5%)	0 (0.0%)	1 (0.6%)	1 (1.4%)	0.682
Perinatal death	4 (2.9%)	3 (4.7%)	3 (1.7%)	3 (4.3%)	0.557
Neonatal death, 0–27 days	4 (2.9%)	1 (1.6%)	3 (1.7%)	3 (4.3%)	0.628
Low Apgar score at 1 min <7	8 (5.8%)	5 (7.8%)	21 (12.1%)	7 (10.1%)	0.287
Low Apgar score at 5 min <7	4 (2.9%)	3 (4.7%)	4 (2.3%)	3 (4.3%)	0.740

Values are *n* (% within age group) unless otherwise indicated. NICU, neonatal intensive care unit; IUFD, intrauterine fetal death. Antep*a*rtum IUFD is a subset of stillbirth and is not an additional mutually exclusive mortality event. Perinatal death comprises stillbirth plus early neonatal death occurring within the first seven completed days of life. Neonatal death comprises death of a liveborn infant during the first 28 completed days.

Preterm birth occurred in 16 of 201 neonates (8.0%) in the younger group and 37 of 243 (15.2%) in the AMA group. The crude OR was 2.08 (95% CI 1.12–3.86), whereas the adjusted estimate was aOR 1.87 (95% CI 0.98–3.56; *p* = 0.057). Across the full cohort, 55 women experienced preterm labour and 53 delivered preterm. Of the 55 women with preterm labour, 50 (90.9%) subsequently delivered before 37 weeks, whereas 5 (9.1%) delivered at ≥37 weeks. Three additional preterm births occurred without recorded preterm labour, which may reflect medically indicated preterm delivery or delivery without a documented episode of preterm labour. Among women aged <35 years, 15 had both preterm labour and preterm birth, 2 had preterm labour followed by term delivery, and 1 delivered preterm without recorded preterm labour. Among women aged ≥35 years, the corresponding counts were 35, 3, and 2, respectively.

Meconium aspiration occurred in 14 of 201 neonates (7.0%) in the younger group and 4 of 243 (1.6%) in the AMA group. The crude association suggested a lower frequency among women aged ≥35 years (OR 0.22, 95% CI 0.07–0.69). Because only four events occurred in the AMA group, no adjusted regression estimate was reported; the finding was considered exploratory and clinically uncertain.

After reconciliation of mortality classifications, nine stillbirths and four early neonatal deaths were identified, corresponding to 13 perinatal deaths. Eleven neonatal deaths occurred within 28 days, including four in the first seven days. No fetal or neonatal mortality outcome showed a statistically reliable age-related association, although event numbers were small.

### 3.5 Secondary grouped comparison

Table 5 presents the raw 2 × 2 cell counts together with crude and adjusted associations. Adjustment for parity, BMI category, and pre-existing diabetes attenuated the estimates for preterm labour (aOR 1.76, 95% CI 0.93–3.31) and neonatal preterm birth (aOR 1.87, 95% CI 0.98–3.56), with both confidence intervals including the null. Outcomes with any exposure-by-outcome cell count <5 were not modelled multivariably because the available information was insufficient for stable adjusted estimation. Continuous-age sensitivity analyses did not materially change the direction of the principal findings. Bonferroni-adjusted post-hoc pairwise comparisons for variables with significant omnibus age-group tests are presented in Table 6.

**Table 5:** Crude and adjusted associations between advanced maternal age and selected maternal and neonatal outcomes.

Outcome	a: ≥35 outcome +	b: ≥35 outcome −	c: <35 outcome +	d: <35 outcome −	Crude OR (95% CI)	Crude p	Adjusted OR (95% CI)	Adjusted p
Preterm labour <37 weeks	38	205	17	184	2.01 (1.09–3.68)	0.029	1.76 (0.93–3.31)	0.081
Preeclampsia	8	235	3	198	2.25 (0.59–8.58)	0.359	Not estimated	—
Antepartum haemorrhage	8	235	8	193	0.82 (0.30–2.23)	0.800	0.75 (0.27–2.09)	0.585
Any caesarean delivery	45	198	43	158	0.84 (0.52–1.33)	0.474	0.91 (0.55–1.50)	0.710
Emergency caesarean delivery	29	214	34	167	0.67 (0.39–1.14)	0.172	0.70 (0.40–1.22)	0.208
Postpartum haemorrhage	3	240	7	194	0.35 (0.09–1.36)	0.197	Not estimated	—
Low birth weight <2,500 g	29	214	15	186	1.68 (0.87–3.23)	0.151	1.51 (0.77–2.96)	0.228
Neonatal preterm birth <37 weeks	37	206	16	185	2.08 (1.12–3.86)	0.019	1.87 (0.98–3.56)	0.057
Meconium aspiration	4	239	14	187	0.22 (0.07–0.69)	0.006	Not estimated	—
NICU admission	14	229	5	196	2.40 (0.85–6.77)	0.103	2.08 (0.73–5.92)	0.169
Low Apgar score at 1 min <7	28	215	13	188	1.88 (0.95–3.74)	0.072	1.66 (0.82–3.35)	0.158
Low Apgar score at 5 min <7	7	236	7	194	0.82 (0.28–2.38)	0.789	0.75 (0.25–2.24)	0.607
Neonatal death, 0–27 days	6	237	5	196	0.99 (0.30–3.30)	1.000	1.01 (0.30–3.42)	0.985

a = outcome present among women aged ≥35 years; b = outcome absent among women aged ≥35 years; c = outcome present among women aged <35 years; d = outcome absent among women aged <35 years. The crude OR was calculated as (a × d)/(b × c). Adjusted ORs were estimated using Firth penalized-likelihood logistic regression controlling for parity, BMI category, and pre-existing diabetes mellitus. Outcomes with any exposure-by-outcome cell count <5 were not subjected to multivariable modelling because the adjusted estimates were considered too unstable for reliable interpretation. Previous caesarean delivery, previous preterm birth, and gestational diabetes mellitus were unavailable for consistent adjustment.

**Table 6:** Bonferroni-adjusted *post-hoc* pairwise comparisons for variables with significant omnibus age-group tests.

**Variable**	**20–29 vs. 30–34**	**20–29 vs. 35–39**	**20–29 vs. 40–45**	**30–34 vs. 35–39**	**30–34 vs. 40–45**	**35–39 vs. 40–45**
**Parity**	1.0000	0.0008	0.0519	0.3579	1.0000	1.0000
**Presenting symptoms**	1.0000	0.0419	0.0252	1.0000	0.4882	1.0000
**Pre-existing diabetes mellitus**	1.0000	1.0000	0.0712	1.0000	0.7224	0.6180
**Abnormal respiratory findings**	1.0000	1.0000	0.6666	1.0000	1.0000	0.4787
**Mode of delivery**	0.1867	1.0000	0.1498	0.1905	1.0000	0.3172
**Any caesarean delivery**	0.1191	1.0000	0.4244	0.0284	1.0000	0.1361
**Meconium aspiration**	1.0000	0.2214	1.0000	0.2070	0.6309	1.0000

Values are Bonferroni-adjusted two-sided *p*-values. Pairwise Pearson chi-square tests were used when expected cell counts were adequate, Fisher's exact test was used for sparse binary outcomes, and the Fisher–Fr*e*eman–Halton exact test was used for sparse multicategory outcomes. Bold values indicate Bonferroni-adjusted *p* < 0.05.

## 4 Discussion

### 4.1 Principal findings

Analysis of the patient-level data from Benghazi Medical Centre showed that advanced maternal age was associated with differences in selected maternal and neonatal outcomes rather than a uniform increase in risk across all outcomes examined. This pattern is broadly consistent with recent global research demonstrating heterogeneity in the magnitude and type of adverse outcomes associated with AMA (12, 13).

When maternal age was dichotomized as <35 vs. ≥35 years, women aged ≥35 years had approximately twice the crude odds of both preterm labour and preterm birth. After adjustment for parity, BMI category, and pre-existing diabetes mellitus, both estimates were attenuated and their confidence intervals included the null, indicating substantial statistical uncertainty. The patient-level analysis nevertheless showed that preterm labour and preterm birth were strongly related: 50 of 55 women with preterm labour subsequently delivered before 37 weeks, while 5 delivered at term and 3 preterm births occurred without recorded preterm labour. This pattern suggests that preterm labour may contribute to preterm birth in many pregnancies, although the present observational analysis cannot establish mediation or causality. Regional studies from Saudi Arabia have similarly reported associations between advanced maternal age and preterm delivery after adjustment for maternal and obstetric characteristics (8–10), supporting the clinical relevance of this pathway while also emphasizing the importance of confounder control.

All cases of pre-existing diabetes mellitus occurred among women aged ≥35 years. This finding is clinically plausible because the prevalence of metabolic disease increases with age; however, pre-existing diabetes represents a baseline maternal condition rather than a complication caused by the index pregnancy. Gestational diabetes mellitus could not be assessed separately because it was not reliably recorded in the source database.

Although the overall distribution of mode of delivery differed among the four maternal-age groups, the grouped analysis did not demonstrate higher overall caesarean delivery among women aged ≥35 years. Adjustment for parity, BMI, and pre-existing diabetes did not materially alter this finding. Nevertheless, interpretation remains limited by the absence of information on previous caesarean delivery, which is likely to be strongly associated with both parity and current mode of delivery.

No statistically conclusive adjusted associations were identified between AMA and antepartum haemorrhage, low birth weight, NICU admission, low Apgar scores, or neonatal mortality. Preeclampsia and postpartum haemorrhage were not subjected to adjusted regression because at least one exposure-by-outcome cell contained fewer than five events. For several outcomes, event numbers were limited and confidence intervals were wide; therefore, the absence of statistical significance should not be interpreted as evidence of equivalent risk between age groups.

Meconium aspiration was less frequent among women aged ≥35 years in the crude analysis, but the AMA group contained only four events and no adjusted model was estimated because of the sparse event count. This finding should not be interpreted as evidence that advanced maternal age is protective. The observed difference may reflect chance, residual confounding, gestational duration, referral patterns, or differences in obstetric intervention and should be regarded as exploratory.

A major strength of the study is its focus on a Libyan obstetric population, for which evidence concerning advanced maternal age remains limited. The use of individual-level maternal and neonatal clinical records permitted assessment of multiple obstetric and neonatal outcomes and allowed crude associations to be distinguished from those persisting after adjustment for important baseline maternal characteristics.

### 4.2 Comparison with prior work

Overall, the present findings support the growing body of evidence suggesting that advanced maternal age is associated with heterogeneous pregnancy risks rather than an inevitable increase in every maternal and neonatal complication (14). Similar patterns have been reported in cohort studies from different healthcare systems, although the magnitude of associations varies considerably across populations and according to the extent of adjustment for maternal characteristics (8, 15, 16).

The crude association between AMA and preterm birth is consistent with previous studies reporting higher frequencies of preterm delivery among older mothers (17–19). Systematic reviews and large observational studies have similarly demonstrated that women giving birth at or beyond 35 years may experience increased risks of both spontaneous and medically indicated preterm birth (4–6). Several mechanisms have been proposed, including higher prevalence of chronic disease, placental dysfunction, increased obstetric intervention, and age-related changes in vascular and uterine function.

Comparable associations have been reported in retrospective cohort studies from China (15, 20, 21). However, the present adjusted analysis demonstrates that the strength of this association may decrease after controlling for important maternal characteristics. This distinction is particularly relevant in the present cohort because multiparity was substantially more common among older women. Parity is independently associated with several obstetric and neonatal outcomes and may therefore confound crude comparisons between age groups (22).

Regional evidence from the Middle East also supports the importance of confounder adjustment. Studies from Saudi Arabia have reported associations between AMA and preterm birth, gestational diabetes, and caesarean delivery, although the magnitude of these associations has varied after adjustment for maternal and obstetric characteristics (8–10). These studies are particularly relevant because pregnancy at advanced maternal age in Middle Eastern populations may frequently occur among multiparous women, contrasting with patterns in some high-income settings where AMA is often associated with delayed first childbirth.

The association between older maternal age and pre-existing diabetes mellitus in the present cohort is consistent with the well-established increase in metabolic disease with advancing age (23). Nevertheless, the inability to identify gestational diabetes separately is important because GDM is one of the most consistently reported metabolic complications of AMA. Its absence from the present dataset may have led to underestimation of age-related metabolic morbidity and prevented evaluation of its potential contribution to outcomes such as fetal growth, preterm birth, and caesarean delivery.

Unlike several previous investigations reporting higher caesarean section rates among older mothers (24, 25), the present study did not identify a significant association when maternal age was analysed dichotomously. Regional studies have also reported increased caesarean delivery among older women (8–10), making the absence of such an association in this cohort noteworthy. Differences in parity, previous operative delivery, referral patterns, clinician decision-making, and local obstetric practice may explain this discrepancy. Because previous caesarean delivery was not consistently recorded, residual confounding by obstetric surgical history remains a major limitation of the delivery-mode analysis.

Variability in the association between AMA and rare adverse neonatal outcomes has been reported in large population-based cohorts (16, 26) and Asian populations (27–29). Abebe et al. (30) found an elevated risk of neonatal morbidity with advancing maternal age; however, some studies have identified less consistent associations or associations limited to specific outcomes. In addition, the absence of a consistent increase in adjusted neonatal morbidity across age groups in the present study indicates that previously reported age-associated morbidity relationships may be influenced by factors including population demographics, baseline maternal health status, obstetric history, and the context in which care was provided.

Although increased risks associated with labor and delivery with advancing maternal age have been reported (31), these associations were not consistently observed in the present study. Differences between the present findings and those of previous studies may reflect variation in population demographics, maternity care pathways, baseline maternal characteristics, and residual or unmeasured confounding rather than necessarily representing contradictory findings.

### 4.3 Interpretation of fetal growth and mortality outcomes

In this analysis, both continuous birth weight and a >4,000 g threshold were used alongside the >4,500 g category available in the existing clinical database. Mean birth weight did not differ significantly across maternal age groups, nor did the proportion of newborns weighing >4,000 g. Only three newborns weighed >4,500 g. Therefore, the small number of newborns meeting the original >4,500 g threshold provided insufficient statistical information to evaluate whether higher maternal age was associated with higher birth weight.

The study used standardized time frames to define fetal and neonatal mortality. Intrauterine fetal death (IUFD) was classified as a subset of stillbirth. Perinatal mortality was defined as stillbirths plus early neonatal deaths occurring within the first seven completed days of life. No statistically significant association was observed between maternal age and fetal or neonatal mortality. However, because of the small number of deaths in the study population, the precision of these analyses was limited; therefore, the findings should not be interpreted as demonstrating equivalent fetal or neonatal mortality risk among older and younger mothers.

### 4.4 Clinical and policy implications

These results are supportive of the idea of developing a personalized prenatal care plan, especially for women ≥35 years old. In making clinical decisions about assessing the risks associated with being pregnant at or over the age of 35, there is reason to evaluate additional factors besides chronological age; these include number of births (parity), presence of pre-existing medical problems, BMI, history of complications during past pregnancies, and specific features of the woman's present pregnancy. Adjustment for multiple variables in the analysis of crude associations shows that AMA needs to be evaluated as part of a comprehensive clinical evaluation rather than separately. This method may enable customized, risk-based follow-up, multidisciplinary care coordination, and informed planning for delivery, similar to what has been found in studies related to pregnancies at older maternal ages (32, 33). Therefore, maternal age ≥35 years should be considered as one component of the overall obstetric risk assessment and should not, by itself, prompt obstetric intervention.

Within the Libyan healthcare system, early identification of women with combined age-related, obstetric, and medical risk factors may facilitate earlier referral, enhanced antenatal monitoring, and timely specialist involvement. Standardized antenatal risk-assessment pathways could incorporate maternal age together with parity, previous caesarean delivery, previous preterm birth, diabetes, hypertension, and other clinically relevant factors when determining the need for secondary or tertiary referral.

At the health-system level, improved routine obstetric data collection is also needed. Standardized recording of gestational diabetes, previous caesarean delivery, detailed parity, previous preterm birth, congenital anomalies, gestational age, and perinatal mortality would strengthen both individual clinical decision-making and national maternal-health surveillance.

The present findings should not, however, be used to estimate national prevalence or to justify population-wide intervention thresholds because the cohort was derived from a tertiary referral hospital. Instead, they identify clinically relevant variables and data gaps that should be incorporated into future multicentre maternal-health research and surveillance in Libya.

### 4.5 Strengths and limitations

A major strength of this study is that it provides individual-level maternal and neonatal data from Libya, where published evidence specifically examining advanced maternal age remains limited. The systematic sampling of records over the defined study period reduced selection associated with specific days or clinical teams. Reviewing original source documents and validating both gestational age and mortality variables helped improve the internal reliability of the data. Although Firth penalized-likelihood logistic regression allows adjustment for covariates in the main outcome analyses, it also reduces small-sample bias. However, it does not provide a complete solution to extreme data sparseness.

There are several other methodological limitations that need consideration. First, this was a retrospective, record-based cross-sectional study at a single center (Benghazi Medical Centre), which prevents causal inferences and limits the generalizability of the findings. Second, because Benghazi Medical Centre is a tertiary referral hospital, the large proportion of women in the sample who were aged ≥35 years indicates possible referral enrichment. Therefore, older or medically complicated women may have been preferentially referred; consequently, the observed frequencies are unlikely to represent the true population prevalence rates in Libya.

Second, parity differed markedly by maternal age. Although parity was included in the adjusted models, only a binary primiparous/multiparous classification was consistently available. More detailed information on number of previous births, grand multiparity, previous preterm delivery, and previous caesarean section was not consistently recorded. Residual confounding by reproductive and obstetric history is therefore likely, particularly for delivery-mode outcomes.

Third, gestational diabetes mellitus was not reliably recorded as a separate variable. Because AMA is an established risk factor for GDM, its omission may have underestimated metabolic morbidity among older women and prevented assessment of its potential confounding or mediating role. Similarly, congenital malformations and chromosomal anomalies were unavailable.

Fourth, several maternal and neonatal outcomes were uncommon. Although Firth penalized regression reduces small-sample bias and problems related to separation, it cannot create information where event counts are very low. Accordingly, outcomes with any exposure-by-outcome cell count <5 were not subjected to multivariable modelling. Even for retained adjusted models, several confidence intervals remained wide; these estimates should therefore be interpreted in terms of their magnitude and precision rather than statistical significance alone.

Fifth, the source dataset defined macrosomia as a birth weight >4,500 g. However, the study also analyzed birth weight >4,000 g and birth weight as a continuous variable to facilitate clinical interpretation of the results. Only three newborns had a birth weight >4,500 g; therefore, the study could draw few conclusions based on this definition.

Sixth, previous caesarean delivery was unavailable. This is particularly important because prior caesarean section is a major determinant of subsequent mode of delivery and may be more frequent among older multiparous women. Consequently, the study cannot determine whether differences in elective or emergency caesarean delivery reflect maternal age, prior obstetric history, or clinical decision-making.

Finally, maternal age was categorized into clinically interpretable groups, which can reduce information compared with continuous modelling. Sensitivity analysis treating maternal age as a continuous variable did not materially alter the direction of the principal findings, but larger datasets would permit more flexible modelling of nonlinear age-related risk without reliance on categorical thresholds.

Future multicentre prospective research with larger and more representative Libyan populations is needed to confirm these findings and to determine the independent contribution of maternal age after comprehensive adjustment for parity, detailed reproductive history, BMI, diabetes, hypertension, previous caesarean delivery, previous preterm birth, antenatal-care characteristics, and other clinically relevant confounders (21).

## 5 Conclusion

Among women aged 20–45 years giving birth at Benghazi Medical Centre from 2017 to 2019, advanced maternal age was associated with higher crude frequencies of preterm labour and neonatal preterm birth, while pre-existing diabetes mellitus occurred only among women aged ≥35 years. After adjustment for parity, BMI category, and pre-existing diabetes mellitus, the associations with preterm labour and neonatal preterm birth were attenuated, and their confidence intervals included the null. Several other outcomes had small event counts and wide confidence intervals, and extremely sparse outcomes were not subjected to adjusted regression. Therefore, the findings do not establish maternal age as an independent causal determinant of the outcomes examined, and the absence of statistical significance should not be interpreted as evidence of equivalent risk between younger and older women.

A key strength of this study is the availability of individual-level maternal and neonatal clinical data from a Libyan setting in which evidence concerning advanced maternal age remains scarce. However, interpretation is limited by the retrospective single-centre design, the tertiary-referral nature of the cohort, the predominance of multiparity among older women, small event numbers for several outcomes, and incomplete measurement of potentially important confounders such as previous caesarean delivery and gestational diabetes.

The results support individualised antenatal counselling and multidimensional risk assessment for women aged ≥35 years, in which maternal age is considered together with parity, pre-existing medical conditions, previous obstetric history, and characteristics of the current pregnancy rather than used as an isolated risk marker. This research provides locally relevant evidence from Libya and highlights the need for multicentre, population-based studies with comprehensive covariate measurement and appropriately adjusted statistical analyses to clarify the independent contribution of maternal age to adverse pregnancy outcomes.

## Data Availability

The raw data supporting the conclusions of this article will be made available by the authors, without undue reservation.
